# A193 DECIPHERING THE INFLUENCE OF CROHN’S DISEASE-ASSOCIATED GENETIC RISK FACTOR, *NOD2* IN INTESTINAL FIBRO-STENOSIS

**DOI:** 10.1093/jcag/gwad061.193

**Published:** 2024-02-14

**Authors:** T Mukherjee, S Patel, J Yadav, A Ayyaz, D Tsang, M Narimatsu, G Bayer, D Trcka, G Bader, J L Wrana, S E Girardin, D Philpott

**Affiliations:** University of Toronto, Toronto, ON, Canada; University of Toronto, Toronto, ON, Canada; University of Toronto, Toronto, ON, Canada; University of Toronto, Toronto, ON, Canada; University of Toronto, Toronto, ON, Canada; University of Toronto, Toronto, ON, Canada; University of Toronto, Toronto, ON, Canada; University of Toronto, Toronto, ON, Canada; University of Toronto, Toronto, ON, Canada; University of Toronto, Toronto, ON, Canada; University of Toronto, Toronto, ON, Canada; University of Toronto, Toronto, ON, Canada

## Abstract

**Background:**

Intestinal fibrosis (IF) is a common debilitating complication associated with Crohn’s disease (CD), an idiopathic inflammatory bowel disease (IBD). IF results in severe strictures that affect the entire gastrointestinal tract, often requiring surgery due to limited or no effective treatment. In general, IF is a consequence of chronic inflammation, excessive extracellular matrix (ECM) deposition, aberrant cellular functions and tissue remodeling involving the innate and adaptive immune system. At the cellular level, fibrosis has been proposed to involve the crosstalk between epithelial cells, mesenchymal stromal cells and immune cells. However, the cascade of events that establish IF is poorly understood as it comprises a dynamic interplay between host genetics, immunity, gut microbiome and aberrant environment insults. Notably, polymorphisms in nucleotide-binding oligomerization domain-containing protein 2 (*NOD2*; a *bonafide* cytosolic innate immune receptor), strong genetic risk factors for CD, also increase the incidence of IF. In this context, how *NOD2* polymorphisms govern intestinal fibrostenosis in CD remains to be investigated.

**Aims:**

To investigate how CD-associated risk gene, *NOD2* contributes to intestinal fibrostenotic disease

**Methods:**

We employed a single-cell RNA sequencing (scRNA-seq) approach to explore the cellular landscape in our CD-mouse model, where we challenged Nod2-deficient mice with a chronic inflammatory insult regime, using dextran sulfate sodium (cDSS) for 3 cycles. Subsequently, changes in inflammatory, metabolic and fibrotic markers in the gut were analyzed by quantitative real-time PCR (qRT-PCR), immunohistochemistry (IHC) or western blot analyses. Further, RNA-In-situ hybridization (RNA-ISH) and flow cytometric analyses were performed to validate cell populations contributing to, and the molecular signatures linked with intestinal fibrostenosis.

**Results:**

We found that Nod2-deficient mice show increased expression of pro-inflammatory cytokines (TNF, IL6) and excessive ECM deposition (fibrosis) in the gut compared to wild-type littermate mice post-cDSS. Curiously, scRNA-seq analysis uncovered altered stromal-immune-cell crosstalk along with the identification of a novel subset of the *Dpt*^*+*^ interstitial stromal population with distinct gene signatures regulated by chronic inflammation. Further, we observed increased senescence (*Cdkn2a*) mediated trans-differentiation of stroma into fibroblast contributing to IF in Nod2-deficient mice.

**Conclusions:**

The study outlined will deepen our understanding of cellular and molecular mediators as well as inter and intra-cellular networks that drive IF. This will be transformative in devising therapeutic strategies to prevent IF in progressive CD patients.

**Funding Agencies:**

CCC, CIHRThe American Association of Immunologist (AAI)

